# Involvement of Autophagy in Coronavirus Replication

**DOI:** 10.3390/v4123440

**Published:** 2012-11-30

**Authors:** Helena J. Maier, Paul Britton

**Affiliations:** Avian Viral Diseases, The Pirbright Institute, Compton Laboratory, High Street, Compton, Newbury, Berkshire, RG20 7NN, UK

**Keywords:** coronavirus, IBV, autophagy, double membrane vesicles, replication-transcription complexes

## Abstract

Coronaviruses are single stranded, positive sense RNA viruses, which induce the rearrangement of cellular membranes upon infection of a host cell. This provides the virus with a platform for the assembly of viral replication complexes, improving efficiency of RNA synthesis. The membranes observed in coronavirus infected cells include double membrane vesicles. By nature of their double membrane, these vesicles resemble cellular autophagosomes, generated during the cellular autophagy pathway. In addition, coronavirus infection has been demonstrated to induce autophagy. Here we review current knowledge of coronavirus induced membrane rearrangements and the involvement of autophagy or autophagy protein microtubule associated protein 1B light chain 3 (LC3) in coronavirus replication.

## 1. Introduction

Coronaviruses are single stranded positive sense RNA viruses belonging to the order *Nidovirales*, and are known to infect a variety of hosts. Several human coronaviruses have been identified, causing mainly mild respiratory infections, with the exception of severe acute respiratory syndrome coronavirus (SARS-CoV). In addition, coronavirus infections have an economic impact on livestock industries worldwide. The avian coronavirus, infectious bronchitis virus (IBV), causes infectious bronchitis (IB), a mild respiratory infection, but as a consequence is responsible for serious effects on the global poultry industries due to poor weight gain in broiler chickens as well as reduced egg production and egg quality in layers. In addition, some strains of IBV are nephropathogenic whilst others result in severe pathology in the reproductive organs. Bovine coronavirus (BCoV) causes respiratory infection and diarrhoea in cattle, transmissible gastroenteritis virus (TGEV) and porcine epidemic diarrhoea virus (PEDV) cause diarrhoea in pigs and porcine haemagglutinating encephalomyelitis virus (PHEV) causes vomiting and wasting disease in pigs.

## 2. Coronavirus Genome Transcription and Replication

Following attachment of coronavirus particles to virus specific receptors on the host cell and uptake of the virus into the cytoplasm, viral genomic RNA is released. This genomic RNA is recognised directly by the host cell translation machinery and two large polyproteins, pp1a and pp1ab, of approximately 400 and 800 kDa are translated. The two polyproteins encode the 15 (IBV) or 16 (all other coronaviruses) non-structural proteins (nsps), which are generated by co- or post-translational cleavage by virally encoded proteases. These proteins assemble into viral replication-transcription complexes (RTCs), providing the virus with the enzymes required for viral RNA transcription and replication, as well as proof-reading and capping of new viral transcripts [1]. In addition, expression of the nsps triggers the rearrangement of host cell membranes, presumed to provide a platform for the assembly of RTCs. It is likely that expression of the membrane associated nsps 3, 4 and 6 is responsible for inducing these rearrangements. Data from closely related arterivirus, equine arterivirus (EAV), demonstrated that expression of nsps 2 and 3 (homologues of nsps 3 and 4 in coronaviruses), in the absence of other viral proteins, was sufficient for the induction of membrane rearrangements and mutation of nsp3 blocked this function [2,3]. Non-structural protein 4 from MHV is known to play a role in formation of rearranged membranes because viruses containing mutant nsp4s show defects in membrane rearrangements and a reduction in virus replication [4,5]. Furthermore, co-expression of nsp4 with the C-terminus of nsp3 from MHV resulted in the relocation of both proteins from diffuse in the cytoplasm to punctate [6]. The authors hypothesised that interaction between these proteins may result in the rearrangement of host membranes [6]. However, the precise mechanism by which the nsps induce membrane rearrangements remains to be elucidated.

**Figure 1:**
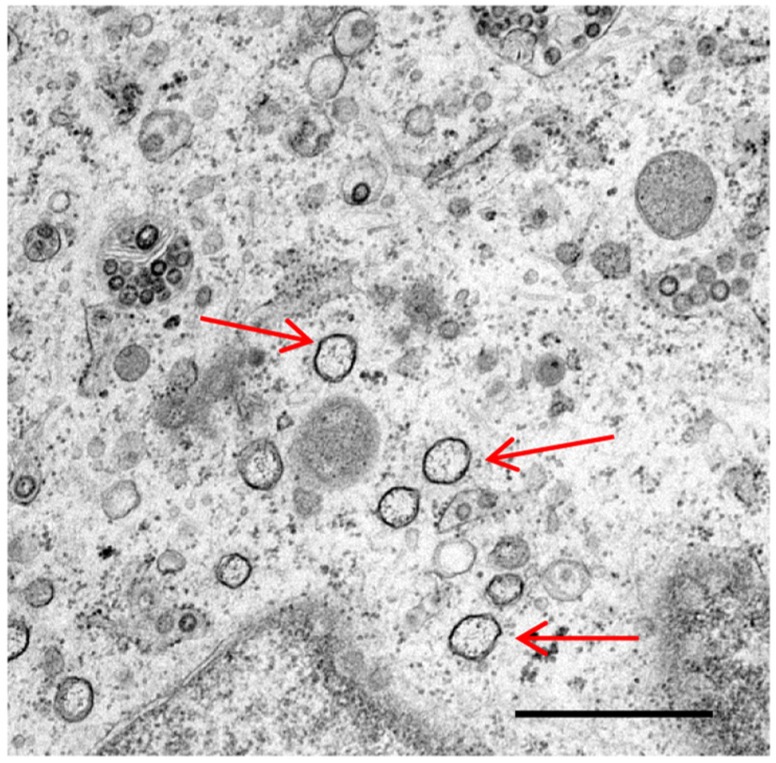
Double membrane vesicles induced by infectious bronchitis virus infection of Vero cells. Vero cells were infected with the Beau-R strain of IBV. Cells were glutaraldehyde fixed at 16 h post infection and prepared for transmission electron microscopy (TEM). Double membrane vesicles are indicated by arrows, scale bar indicates 1 µm.

The nature of membrane rearrangements has been well studied in both SARS-CoV and MHV infected cells by conventional transmission electron microscopy or by electron tomography. Both viruses have been shown to induce double membrane vesicles (DMVs) as well as regions of convoluted membranes [7,8,9]. Electron tomography data showed that the convoluted membranes in SARS-CoV infected cells were derived from and joined to the rough ER. In addition, the outer membranes of the DMVs were interlinked, studded with ribosomes and were also joined to the convoluted membranes and ER. Openings between the interior of the DMVs and the cytoplasm were not observed [7]. In contrast, ribosomes were not seen on the membranes of MHV induced structures [8]. Preliminary work presented here (Figure 1) shows for the first time that IBV infection of mammalian Vero cells also results in the induction of DMVs. However, a detailed analysis of the membrane rearrangements triggered by IBV infection of mammalian and avian cells remains to be performed.

The site of assembly of coronavirus RTCs is currently unclear. During the process of transcription and replication of the viral genome, both positive and negative sense RNAs are synthesised. As a result, dsRNA can form, possibly as a replicative intermediate. This dsRNA has been used as a marker for sites of viral RNA synthesis. However, in SARS-CoV infected cells, dsRNA was found to predominantly locate on the interior of DMVs while the majority of the nsps were found to locate on the convoluted membranes. High resolution immunofluorescence microscopy also demonstrated a separation in the signals for nsps and dsRNA. In addition, due to the lack of any connecting channels between the interior of DMVs and the cytoplasm, questions were raised about transport of RNA to sites of virus assembly at the ERGIC [7]. Subsequent work using 5-ethynyl uridine (EU) to label nascent RNA demonstrated a degree of co-localisation between EU and dsRNA signals at earlier time points of infection but this co-localisation was significantly reduced at later time points [10]. Therefore, further evidence is required to determine the precise location of coronavirus RTCs and the site of RNA synthesis.

## 3. Autophagy

**Figure 2:**
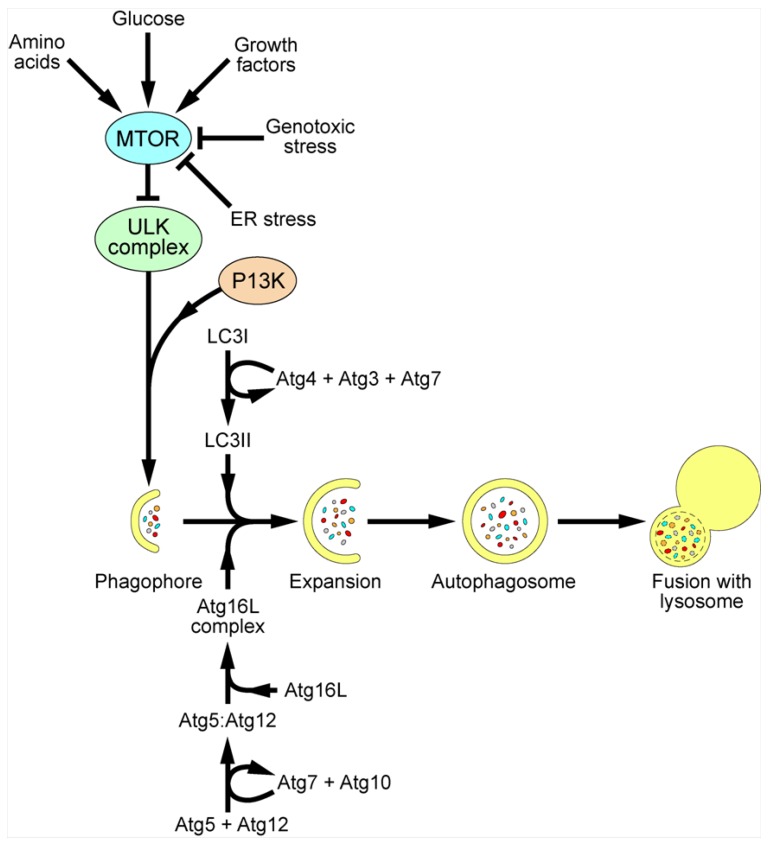
Schematic of mammalian autophagy pathway. MTOR is the major control complex for autophagy. MTOR senses levels of amino acids, glucose and growth factors, as well as genotoxic and ER stress. Upon stimulatory signals, MTOR becomes inactivated and the ULK complex becomes hypophosphorylated and relocalises to the phagophore, along with PIP3, produced by class III PI3K complexes. The Atg16L complex and LC3II are also recruited to the growing autophagosome, allowing expansion of the membrane and fusion to give a complete autophagosome, engulfing organelles, aggregated proteins and intracellular pathogens. The autophagosome then fuses with a lysosome, resulting in the degradation of the contents and recycling of nutrients into the cytoplasm.

Autophagy is a cellular pathway for self-degradation. The pathway allows a cell to degrade long-lived proteins, aggregated proteins and organelles during periods of starvation to provide nutrients for continued cellular processes, as well as playing an important role in cellular homeostasis, ageing and development [11,12,13,14,15]. In addition, dysregulation of autophagy plays an important role in the development of some cancers [16]. During autophagy, regions of the cytoplasm become engulfed into double membrane bound vesicles termed autophagosomes. These vesicles then fuse with late endosomes/lysosomes, where the contents are degraded by lysosomal proteases (Figure 2). For detailed reviews of autophagy signaling, see [17,18]. The major control complex for autophagy is MTOR (mammalian target of rapamycin) which, when active, inhibits initiation of the pathway. MTOR senses amino acid levels, as well as levels of growth factors and glucose and genotoxic and ER stress. Under resting conditions, MTOR phosphorylates and inactivates the ULK complex, comprising unc-51-like kinase 1/2 (ULK1/2), focal adhesion kinase family-interacting protein of 200 kDa (FIP200) and mammalian ATG13. Under stimulatory conditions, MTOR is inactivated; the ULK complex becomes hypophosphorylated and relocates to the site of formation of the autophagosome, the phagophore. Formation of the autophagosome proceeds by addition of new membrane to the phagophore, as opposed to budding from an existing membrane, in a poorly understood process. However, a number of proteins are known to be required. Recruitment of these proteins occurs via the generation of phosphatidylinositol 3-phosphate (PI3P) by the class III phosphatidylinositol 3-kinase complex (PI3K), including BECN1 (Beclin 1). Inhibition of PI3K activity using drugs such as wortmannin and 3-methyladenine (3-MA), and sequestration of BECN1 by antiapoptotic protein B-cell lymphoma/leukemia-2 (Bcl2) all inhibit autophagy. 

Elongation of the autophagosome membrane and formation of the complete autophagosome requires the recruitment of 2 ubiquitin-like (Ubl) conjugation systems. In the first, ATG12 becomes conjugated to ATG5 in a process requiring the E1-like enzyme ATG7 and the E2-like enzyme ATG10. ATG12-ATG5 then binds to ATG16L and forms a large complex known as the ATG16L complex. This complex localises to the phagophore and can determine the site of conjugation of the second Ubl system. In this second system, microtubule associated protein 1B light chain 3 (LC3) is initially cleaved by ATG4 near the C-terminus at position G120 to generate cytoplasmic LC3-I. This subsequently becomes lipidated with phosphatidylethanolamine (PE) in a process requiring ATG7 and another E2-like enzyme ATG3 to generate membrane tethered LC3-II [19]. LC3-II is inserted into both the inner and outer membranes of the autophagosome, and as such, remains associated with the autophagosome throughout the pathway [20,21]. As a result, LC3 has become an extremely valuable marker protein for studying autophagy.

In addition to its role in cellular homeostasis, autophagy has been shown to have a function in innate immunity by degrading intracellular pathogens, including viruses. Furthermore, autophagy plays a role in presenting pathogen components to the immune system [22,23,24,25]. Inhibition of autophagy has a positive effect on the replication or virulence of herpes simplex virus 1 (HSV1) [26,27], Sindbis virus [28,29] and vesicular stomatitis virus (VSV) [30]. In addition, the capsid protein of Sindbis virus was found to be specifically targeted to the autophagosome via an interaction with the autophagy cargo receptor, p62 [28]. However, many viruses have evolved mechanisms to evade autophagy by inhibiting the pathway, or diverting the process to benefit virus replication. Many viral proteins have been identified that inhibit formation of autophagosomes. For example, HSV1 protein ICP34.5 binds to BECN1 and inhibits autophagosome formation and Kaposi’s sarcoma associated herpesvirus (KSHV) and murine γ-herpesvirus (MHV-68) encode Bcl2 homologues to bind to and inhibit BECN1 [26,31,32]. KSHV also encodes another protein to block LC3 processing by inhibiting ATG3 [33]. Other viruses have developed mechanisms to inhibit fusion of autophagosomes with lysosomes. Human immunodeficiency virus (HIV) encoded protein Nef interacts with BECN1, inhibiting lysosomal fusion [34]. Influenza A virus protein M2 has also been shown to induce accumulation of autophagosomes as a result of inhibition of lysosomal fusion, possibly via an interaction with BECN1 [35]. Moreover, numerous viruses have been identified which require autophagy for optimal replication, including hepatitis B virus (HBV) [36], poliovirus [37], coxsackievirus, HIV-1 [38], hepatitis C virus (HCV) [39], Dengue virus [40,41] and Japanese encephalitis virus [42]. Finally, poliovirus subverts autophagy in order to generate membranous structures required for assembly of viral replication complexes [37,43]. For more detailed information about the role of autophagy in the replication cycles of viruses, see reviews [44,45,46,47,48,49].

## 4. Coronavirus Replication and Autophagy

The presence of DMVs in coronavirus infected cells suggested that this group of viruses might, like other positive sense RNA viruses, utilise the autophagy pathway to generate the membrane structures required for replication. Initial work showed that MHV infection induced autophagy [50]. Nsp8 co-localised with LC3 throughout infection and the nucleocapsid protein N co-localised with LC3 early in infection, but this decreased over time. In addition, MHV replication was markedly reduced in ATG5^−/−^ embryonic stem cell lines but virus titre was rescued in the presence of an ATG5 expressing plasmid [50]. Further work using SARS-CoV again showed co-localisation between nsp8 and LC3 [51]. However, work by others using bone marrow derived macrophages lacking ATG5 or ATG5^−/−^ primary murine embryonic fibroblasts (MEFs), showed that MHV replication does not require either an intact autophagy pathway or conversion of LC3I to LC3II. They did however confirm that SARS-CoV nsps co-localised with LC3 [52]. Recently, Schneider et al. demonstrated that SARS-CoV replication could also occur in ATG5^−/−^ MEFs, indicating no requirement for a complete autophagy pathway [53]. Interestingly, it was observed that virus replication was unaffected by the induction of autophagy in wild type MEFs [53]. Finally, de Haan *et al*. were unable to show co-localisation between MHV nsp8 and GFP-LC3 [54], and Snijder *et al*. were unable to show co-localisation between SARS-CoV nsp3 and either endogenous LC3 or GFP-LC3A, GFP-LC3B or GFP-LC3C [9]. Despite the lack of clarity with regard to the requirement for autophagy during coronavirus infection, all studies did indicate that LC3 became punctate upon coronavirus infection, suggesting an induction of autophagy [9,50,51,52,54]. 

Consistent with previous observations, recent experiments performed in mammalian cells using IBV showed that this avian coronavirus is also capable of inducing autophagy during infection [55]. Here, further work was performed and individual expression of viral nsp6 was also capable of inducing autophagy, whereas nsp4 and nsp10 were not. Nsp6 induced autophagosomes fused with LAMP1 labelled lysosomes and autophagosomes were susceptible to wortmannin treatment. This indicates that bone fide autophagy was induced. Interestingly, nsp6 homologues from SARS-CoV, MHV and arterivirus porcine reproductive and respiratory syndrome virus (PRRSV), also induced autophagy. The mechanism for the induction of autophagy was not determined, although it was shown not to be by induction of ER stress, MTOR inhibition or via sirtuin 1. In addition, it was not confirmed whether nsp6 was responsible for the induction of autophagy in virus infected cells. However, in agreement with previous work, virus infection was not inhibited by the knockdown of ATG5 expression [55].

In other work, it has been suggested that whilst endogenous LC3 could co-localise with MHV RTCs, GFP-LC3 showed significantly reduced co-localisation [56]. This may provide an explanation for some of the earlier experimental discrepancies. Further work in this study demonstrated that LC3 puncta were still observed in MHV infected ATG7^−/−^ cells, where LC3I to LC3II conversion cannot occur and also in cells expressing LC3 that cannot be processed to LC3II (LC3 G120A). In addition, although the absence of the complete autophagy pathway due to a lack of ATG7 did not alter MHV replication, reduced expression of LC3 by RNAi did significantly reduce virus replication. Furthermore, viral protein expression could be rescued in the presence of LC3 G120A [56]. This demonstrated that MHV replication does not require the complete autophagy pathway, or conversion of LC3I to LC3II, in agreement with previous work, but instead required LC3I [52]. Interestingly, MHV RTCs co-localise with markers for cellular EDEMosomes [56,57], vesicles involved in ER associated protein degradation (ERAD) tuning and knockdown of EDEMosome cargo receptor SEL1L reduced virus replication [57,58]. In the ERAD pathway, unfolded proteins are removed from the ER and targeted for degradation. However, under normal conditions, the ERAD machinery must be regulated to prevent premature removal of proteins before they have been folded [59]. During ERAD tuning, parts of the ERAD machinery are removed from the ER in EDEMosomes to down regulate the pathway [57,58,60]. LC3I is recruited to EDEMosomes by transmembrane protein SEL1L [57] and possibly acts as a coat protein [60]. This work has highlighted a role for LC3 as part of the ERAD tuning pathway, but not autophagy, in the replication of MHV. Whether this pathway is involved in the replication cycles of other coronaviruses remains to be determined.

## 5. Future Questions and Perspectives

Although mounting evidence suggests that autophagy is unlikely to play a role in the replication of coronaviruses and the generation of coronavirus replicative structures, several questions remain unanswered. Work performed so far focusses mainly of members of the betacoronaviruses, MHV and SARS-CoV. Detailed characterisation of the membrane structures induced in cells infected with alphacoronaviruses, like transmissible gastroenteritis virus (TGEV), or gammacoronaviruses, like IBV, needs to be performed. Although these viruses are related, it is possible that there are differences in the mechanisms of membrane rearrangement and the types of structures induced. In addition, the location of RTC assembly and the site of viral RNA transcription and replication need to be identified. The membranes induced in MHV and SARS-CoV infected cells are complex and how the different structures play a role in virus replication is currently unknown. Furthermore, the mechanism by which these rearrangements are generated is not understood. Whether MHV induced membranes are altered upon inhibition of ERAD tuning and which proteins might be involved in hijacking the pathway remains unknown. Moreover, whether this pathway is important for the replication of other coronaviruses is also unknown.

Recently the replication of IBV has been shown to induce autophagy in mammalian cells [55]. Furthermore, individual expression of IBV nsp6, as well as nsp6 homologues from other viruses in the *Nidovirales* order, has been shown to induce autophagy [55]. However, the mechanism by which this induction occurs is unknown. Whether nsp6 is responsible for inducing autophagy in the context of whole virus would also be interesting to discover. In addition, as IBV is restricted to avian species, would similar observations be made in a more natural host model? Finally, several studies have shown that coronavirus infection induces autophagy [9,50,51,52,54,55]. However, the pathway does not appear to be required for virus replication [52,53,55,56]. Therefore, is autophagy acting as a cellular defence to virus infection and does the virus have mechanisms to control this response?
